# A Computerized Prediction Model of Hazardous Inflammatory Platelet Transfusion Outcomes

**DOI:** 10.1371/journal.pone.0097082

**Published:** 2014-05-15

**Authors:** Kim Anh Nguyen, Hind Hamzeh-Cognasse, Marc Sebban, Elisa Fromont, Patricia Chavarin, Lena Absi, Bruno Pozzetto, Fabrice Cognasse, Olivier Garraud

**Affiliations:** 1 GIMAP-EA3064, Université de Lyon, Saint-Étienne, France; 2 Laboratoire Hubert Curien - UMR CNRS 5516, Saint-Etienne, France; 3 EFS Auvergne-Loire, Saint-Etienne, France; University of Leuven, Rega Institute, Belgium

## Abstract

**Background:**

Platelet component (PC) transfusion leads occasionally to inflammatory hazards. Certain BRMs that are secreted by the platelets themselves during storage may have some responsibility.

**Methodology/Principal Findings:**

First, we identified non-stochastic arrangements of platelet-secreted BRMs in platelet components that led to acute transfusion reactions (ATRs). These data provide formal clinical evidence that platelets generate secretion profiles under both sterile activation and pathological conditions. We next aimed to predict the risk of hazardous outcomes by establishing statistical models based on the associations of BRMs within the incriminated platelet components and using decision trees. We investigated a large (n = 65) series of ATRs after platelet component transfusions reported through a very homogenous system at one university hospital. Herein, we used a combination of clinical observations, *ex vivo* and *in vitro* investigations, and mathematical modeling systems. We calculated the statistical association of a large variety (n = 17) of cytokines, chemokines, and physiologically likely factors with acute inflammatory potential in patients presenting with severe hazards. We then generated an accident prediction model that proved to be dependent on the level (amount) of a given cytokine-like platelet product within the indicated component, e.g., soluble CD40-ligand (>289.5 pg/109 platelets), or the presence of another secreted factor (IL-13, >0). We further modeled the risk of the patient presenting either a febrile non-hemolytic transfusion reaction or an atypical allergic transfusion reaction, depending on the amount of the chemokine MIP-1α (<20.4 or >20.4 pg/109 platelets, respectively).

**Conclusions/Significance:**

This allows the modeling of a policy of risk prevention for severe inflammatory outcomes in PC transfusion.

## Introduction

Transfusion is a safe process and leads to few adverse events (AEs), especially because systematic leukoreduction was implemented for all labile blood components (LBCs). In addition, platelet component (PC) transfusions induce, in general, three times more AEs than red blood cell component (RBCC) transfusions. Platelet-associated AEs occur in 1 out of every 1030 PC transfusions [1]. Despite residual leukocyte links, AEs cannot be completely eliminated [2]. There are evidence-based observations that the factors associated with stored platelets themselves play a significant role in those AEs, especially in the most severe ones, termed acute transfusion reactions (ATRs) [3]–[5]. These factors include a large number of microparticles; oxygenated moieties of membrane lipids; inflammatory mediators such as histamine, serotonin, and ADP/ATP; and biological response modifiers (BRMs) that themselves comprise cytokines, chemokines, growth factors, inhibitory factors, and related molecules [2], [6]–[11]. There is further evidence that all pro-inflammatory factors increase over time (at 22±2°C, in general up to 5 days and occasionally up to 7 days) in stored PCs, which constitute the transfusion inventory issued to patients in need [12], [13]. BRMs in particular are principally shed from the platelet membranes or secreted from docks [14], [15]. Soluble CD40-Ligand (sCD40L), also known as sCD154, is considered to be the master pro-inflammatory mediator secreted by platelets [16], [17]. In transfusion pathology, some donor platelet a granules are emptied of sCD40L, and almost all secreted factors are freed in the PC supernatant [18]. Some other BRMs are known to be associated with platelets: some were acknowledged to be platelet factors among the already 300 known ones [18], [19], whereas others were later recognized as platelet factors because they were found in transfusion pathology [20]–[22]. Despite these findings, relating the presence or the increased level of a given BRM within the issued PC and an ATR outcome in the recipient is difficult because all cases address reported hazards and do not overview the non-hazardous events. Additionally, the BRM levels are measured in pathological cases despite elevated concentrations of putatively noxious factors that may also be found in PCs considered safe. Finally, most relationships established thus far between potentially harmful BRMs (from the donor's blood) and recipients presenting with ATRs have been determined by means of *ex vivo* assays, with neither direct proof of significance nor clinical relevance in the donors or patients. To overrule this caveat, we sought to collect clinical information regarding PC-linked ATRs and the residual PCs administered to these patients, which were shipped to our laboratory, and to measure a large variety of BRMs, which were then compared with asymptomatic pairs of recipients and PCs. This strategy allowed the identification of 14 relevant BRMs, with some being expected (such as sCD40L) and others not previously associated with platelets; it further allowed the creation of profiles of BRMs linked with clinical presentations; last, it permitted the establishment of predictive models of hazard outcome based on levels of sCD40L, IL-13, and MIP-1α. This approach, combining clinical reports, biological data, and mathematical/statistical models, is original and may help develop predictive tests to prevent the transfusion of possibly harmful PCs, especially in fragile patients unable to cope with inflammatory conditions.

## Materials and Methods

### Cases and controls

We reported previously on the methods for collecting single donor apheresis (SDA)-PCs at our blood establishment (BE) (See Methods S1). Briefly, apheresis platelets were collected from regular anonymous blood donors (Regional Blood Bank, EFS Auvergne-Loire - http://www.dondusang.net) who volunteered to provide blood for research purposes and signed a consent form, approved by the ethical committees of Etablissement Français du Sang [23]. PCs are identified with bar-codes and none of the investigators can reconcile any single Donor and his/her given BC (only the Blood service physician can in case of control sampling is needed for the Donor, regarding a potential infectious risk). Further, Recipients' data are anonymized with Hospital attributed bar-codes. None of the authors can access the patient's file. All needed data is provided anonymously by the physician in charge. Thus, this study is completely anonymized. The transfusions were conducted as part of routine care in the close-by University Clinics; the clinics' physicians was in charge to report on the AEs/ATRs, to describe the symptoms and to forward the PC identification number, along with a PC sample if some was left-over. The investigators' role here was 1) to design the study, 2) to educate clinicians and nurses in clinics to report on AEs to them as well, and 3) to educate Labwork technicians to save the bags (shipped back to them according to the procedures in force) and to ship them to the Laboratory Research facilities. All samples harbored the Hospital bar-code # to identify the recipient, but in no way the clear identification of the patient in this case. This procedure protects the anonymity, according to the French Regulation (CNIL).

We identified 65 PCs that were associated with ATRs during the years 2008 to 2011 from over more than approximately 23,250 SDA-PCs produced and delivered during this period. Only patients who received only one blood component (BC)—for instance a PC—in the current transfusion episode were enrolled in the present survey; this measure was taken in order to make sure that the considered ATR was indeed related to the BC (PC); this does not preclude that other BCs were not transfused prior to the last PC having led to the recorded ATR, but when that was the case, the last transfused BC was given at least 16 h before (16 h being considered the maximum timeframe to rely an ATR to e.g. the occurrence of TRALI due to a given BC, according to the majority of published consensuses). Furthermore, several PCs that were associated with ATRs were not included and interpreted in this study. Patients with previously known allergic cases (ATRs) were discussed from the present study, as now stated in the Material & Methods section.

In ATR cases, the remainders of the PC bags were shipped back to the BE facility, along with patient serum samples, for further investigation by split sampling: one sample was used to investigate the possibility of TTBI, another one to examine the parameters of blood compatibility (immune hematology), and a third one to test the inflammatory markers (this study). The PCs were transferred in a polypropylene tube and centrifuged at 500 g for 15 minutes. The supernatants were stored at −80°C for the soluble factor assays. The 65 PCs that were associated with ATRs were compared with 59 control PCs that were not associated with an ATR. The two sets of PCs were matched in terms of storage duration.

All considered cases were scored as 3 (severe) according to the ISBT scaling system [24], i.e., necessitating medical assistance, with no grade 4 (i.e., lethal) cases observed in this survey. The cases with accountability grades of 3 (“probable”) and 4 (“certain”)—in terms of accountability according to this international scale—were retained for the survey, and the “unlikely” and “possible” cases were discarded.

After having excluded hazards obviously linked to the causal pathology in the transfused patients as well as the infectious (TTBI) causes, the diagnosis of inflammatory-type ATR was made on the immediate observation/report of 1) FNHTR, generally associated with fever, rigors, and/or chills; 2) AATR, which commonly involves erythematous rash, urticaria, and/or pruritus or more severe reactions with angioedema, which are combined with the further discharge of typical allergic biology, such as elevated serum tryptase, histamine, or IgE; and 3) in rare occasions, HT with tachy-/bradycardia and/or hyper-/hypotension resembling non-septic shock. Those pathologies are, in general, associated with the inflammatory cases [25]. We excluded infectious shock, overload, and objective cardiopulmonary lesions. We also excluded all cases of ATR with a known Ag/Ab conflict such as allo-immunization (against the donor's HLA and/or HPA), post-transfusion purpura, or refractoriness (and bleeding).

For the present study, we selected 59 “control” APCs that were matched with each ATR sample for the same storage time, preparation processor, and PAS and did not induce any ATR. These controls were randomly included in the storage group. The control SDA-PCs were also matched for the gender and age of the donors (**Table 1**).

**Table 1:** Parameters of platelet donors and storage time of the platelet concentrates.

Group			Control (n = 59)	Adverse effects (n = 65)
**Sex** a	Male		61	60
	Female		39	40
**Age (years - mean ± Std)**			47.25±12.58 (21–67)	50.95±8.77 (27–65)
**Storage time** a **(days)**	<3		25.42	20
	≥3		74.58	80

a The data are shown as percentages.

### Cytokine, chemokine, and biological response modifier measurement in platelet component supernatants

PCs that resulted in ATR were shipped almost immediately to the BE's research facilities. The supernatants were discarded within hours and frozen until assay, with a maximum of 12 h of elapsed time in the case of night AE (**Fig. S1**).

With the exception of certain BRMs for which the Luminex format is either not available or not convenient, we mainly focused on factors that could be tested by highly reproducible technology. We thus tested Gro-α, sCD40L, 6-Ckine (CCL21), CXCL9, IL-13, IL-15, IL-23, IL-33, MIP-1α, IFNγ, MDC, CCL19, CCL20, BCA-1, and TSLP in the PC supernatants using Luminex technology (using panels I, II, and III: HCYTOMAG-60K-08, HCYP2MAG-62K-05, and HCYP3MAG-63K-03; Millipore, Molsheim, France), according to the manufacturer's instructions. The results were determined using a Bioplex 200 system (BioplexManager software; Biorad, Marnes-la-Coquette, France) and adjusted to 10^9^ platelets.

Alternatively, RANTES and sCD62P were tested via ELISA using a commercial kit (R&D Systems Europe Ltd, Lille, France) according to the manufacturer's instructions and as described previously [2]. Duplicate ELISA data for each sample were fitted separately and then averaged to provide the final result and standard deviations. Absorbance at 450 nm was determined with an ELISA reader (Magellan software Sunrise; Tecan group Ltd., Lyon, France). Data (expressed in pg/ml) were adjusted to 10^9^ platelets.

### Statistics

The concentrations of soluble factors between the two groups were compared using a two-tailed Student's t test, and ANOVA tests were performed to compare these concentrations per storage day. For each factor, the difference was considered significant if the p-value was <0.05. Correlations between the variables were assessed using Pearson coefficients. One given correlation was considered significantly different from zero when the p-value was <0.05.

Receiver operating characteristic (ROC) curves were used to determine the cutoff values of soluble factor assays, and the AUCs were used to calculate the discriminatory ability of every candidate factor and classify them as potential sources of ATR. For each factor, a two-sample z-test was performed to test the null hypothesis. If the calculated p-value was below the significance level (α = .05), then the AUC was considered significantly different from 0.5 (null hypothesis, meaning no discriminating power). All statistics were calculated using computer software XLSTAT (Addinsoft, Paris, France).

### Decision-tree learning

Decision-tree learning, used in statistics, data mining and machine learning, uses a decision tree as a predictive model that maps observations about an item to conclusions about the item's target value. Decision-tree learning aims to predict the value (called the class) of a particular target attribute for unseen data (called the test set), according to the values of other attributes for known examples (called the training set). The internal nodes of the tree represent tests on a given attribute, each branch represents the outcomes of this test, and each leaf node represents the class label (the decision taken). A path from the root node to a leaf can be viewed as a classification rule. The general “Top Down Induction of Decision Tree” (TDIDT) algorithm is given as follows:

**Function** TDIDT(E: set of examples) **returns** tree;

T': =  grow_tree(E);

T : =  prune(T');

**return** T.

**Function** grow_tree(E: set of examples) **returns** tree;

T : =  generate_tests(E);

t : =  best_test(T,E);

P : =  partition induced on E by t;

if stop-criterion(E,P) then **return** leaf(info(E)).

**else** **for** all Ej in P: tj : =  grow_tree(Ej);

**return** node(t, {(j, tj)});

The algorithm first “grows” a tree and then possibly “prunes” it to address potential over-fitting phenomena using the training set (using error rates or statistical error pruning based on a minimum description length principle). The main “Grow_tree” function has some general characteristics that vary according to the particular chosen algorithm. We chose the most well-known decision tree learning algorithm, known as C4.5, and implemented it in the Weka platform (Weka, University of Waikato, New Zealand) [26]. For continuous attributes, C4.5 generates as many tests as possible to separate between two consecutive values of this attribute in the training set. For discrete attributes, all the possible tests are generated. To select the best test, C4.5 makes a decision based on maximizing the gain brought by each test to the global entropy computation based on the set of examples involved in this test. The stopping criterion is a statistical test based on a minimum description length principle (herein, we used the default value provided by Weka, which was 0.25). The class label in each leaf is the majority class of all training examples for which all tests from the root to this leaf are true.

Decision-tree learning algorithms are popular algorithms in machine learning because they produce sound (it generalizes standard statistics), simple, and interpretable prediction models.

In our particular case, from a set of 101 training samples with 17 attributes (age of the blood sample, age of the donor, platelet count, and levels of Gro-α, sCD40L, 6-Ckine, CXCL9, IL-23, MIP-1α, IL-13, IFNγ, IL-15, MDC, IL-33, CCL19, CD62P, and RANTES), we could predict the risk associated with a given cytokine if present, absent, or present in excess (3 classes: AATRs, FNHTRs, and Control), which allows the forecasting of unfavorable outcomes in patients.

## Results

### The study population

The study population consisted of patients who received single donor apheresis (SDA)-PC transfusions in the previous 3 years in a very homogeneous case observational study: one single blood establishment (BE) collected, processed, prepared, controlled, and transfused the PCs and contributed to the follow-up (surveillance) of the cases. The surveillance, however, of the transfused patients was conducted by one dedicated team of physicians in a single university hospital, who agreed to discuss the cases with the BE to harmonize the declaration of an AE in accordance with the national regulation in force. Over the investigation period, 65 inflammatory-type ATRs were reported, which excluded obvious or later acknowledged cases of bacterial infections TRALI, severe and documented allergy cases, and cases obviously linked to an Ag/Ab conflict. The parameters linked to the PCs (65 “pathogenic” and 59 “non-noxious”) are presented in Table 1. There were no significant differences in the patient-dependent parameters. Furthermore, after dividing the ATR and control populations by donor age into five arbitrary categories (<30, 31–40, 41–50, 51–60, and >60 years of age), we identified no significant association between donor age and ATR occurrence in the recipients (χ^2^ test >0.05). The sex ratios in the control- and ATR-associated donors were similar (1.56 and 1.5, respectively; z test >0.05). Thus, there was no association between the gender of the donors and ATR occurrence in the recipients (OR = 0.96, χ^2^ test >0.05).

We next attempted to examine whether there was a relationship between the mean age of the platelets at delivery and the broad clinical presentation. We considered three main categories of clinical presentations; however, these categories do not obey a strict consensual (international) classification as we considered them broadly “inflammatory type.”

**Figure 1:**
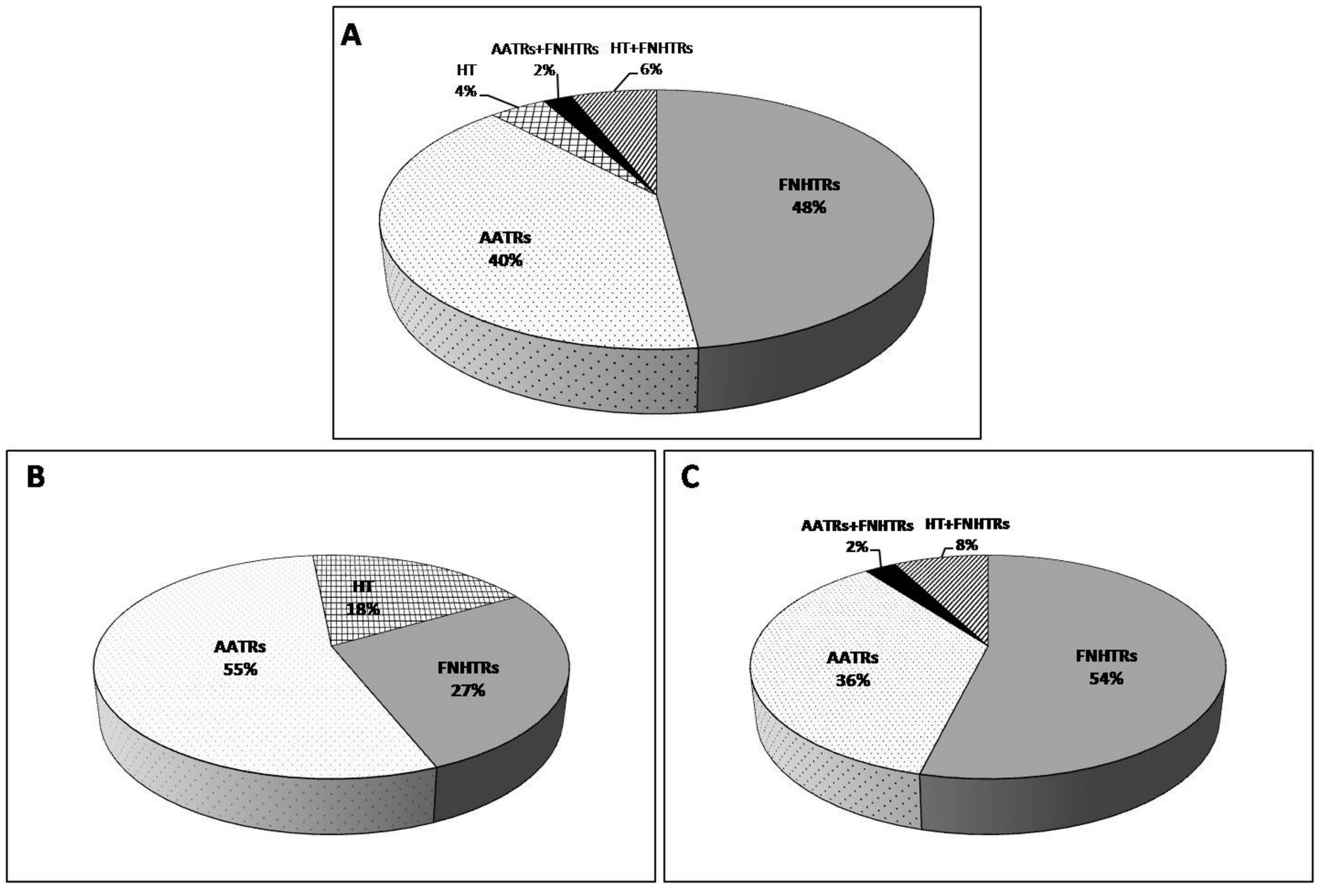
Distribution of AE clinical observations resulting from a platelet transfusion. **A**. All PCs. **B**. PCs delivered before 3 days. **C**. PCs delivered from 3 to 5 days. The data are shown as percentages. FNHTR, febrile non-hemolytic transfusion reaction (fever or chill); AATRs, atypical allergic transfusion reactions (erythematous rash, urticaria, and/or pruritus or more severe reactions with angioedema); hemodynamic trouble (HT), excluding ALI (and TRALI), TACO, myocardial infarctions, and pulmonary embolism; combined ATRs, ATRs with two or more associated manifestations. We did not analyze any case with bronchospasm or anaphylaxis.

ATRs (n = 65) were divided as follows: febrile non-hemolytic transfusion reactions (FNHTRs), 48%; atypical allergic transfusion reactions (AATRs), 40%; and hemodynamic trouble (HT) (excluding ALI [and TRALI], transfusion-associated circulatory overload [TACO], myocardial Infarction, and pulmonary embolism), 4% (**Fig. 1A**). This classification system is in line with what is usually used in hemovigilance reporting systems [1]. The ATR distribution was different when PCs were delivered before (20%) (**Fig. 1B**) or after 3 days (80%) (**Fig. 1C**) of age (shelf-life). Then, we investigated each population before or after the 3-day PC storage period. We observed that in the ≤3-day storage group, the majority of reported ATRs were AATRs (55%), followed by FNHTRs (27%) and HT (18%). In the >3-day group, we observed that the majority of reported ATRs were FNHTRs (54%), followed by AATRs (36%) and HT (10%). FNHTRs were significantly less frequent (27% vs. 54%; z test, p = 0.017), whereas AATRs were more frequent (55% vs. 36%; z test, p = 0.269) when the PCs were “fresher”.

The 3-day threshold was considered based on our findings regarding PC storage time and the secretion of significant amounts of pro-inflammatory cytokines, particularly those that lead to ATRs (20% vs. 80% in the <3-day vs. 3–5-day storage periods, respectively, p<0.05) (**Table 1**).

As PCs were obtained with two distinct types of processes, we sought to examine the possibility that one process activates platelets more than the other with respect to the secretion of soluble, inflammatory-type factors. No consistent difference between the Amicus and Trima processes was observed, with the exception of a higher Gro-α concentration on days 3 and 4 in PCs prepared with the Amicus system and higher CCL19 concentration in PCs prepared with the Trima system; however, these variations were homogenized by day 5 (**Fig. S2**). The data were also similar regarding the platelet additive solutions (PAS) used (data not shown). In aggregate, we found no differences in the soluble factors in the PCs that produced ATRs between the two processes.

### Cytokines and related secreted factors found in left-over PCs that produced ATRs

The supernatants of SDA-PCs that resulted (65) or not (59 matched controls) in inflammatory-type ATRs were tested for 17 BRMs, which were available for analysis using the Luminex platform. sCD40L was tested as a reference marker because of its consistent association with ATRs [6], [15], [17], [27]–[29].

**Figure 2:**
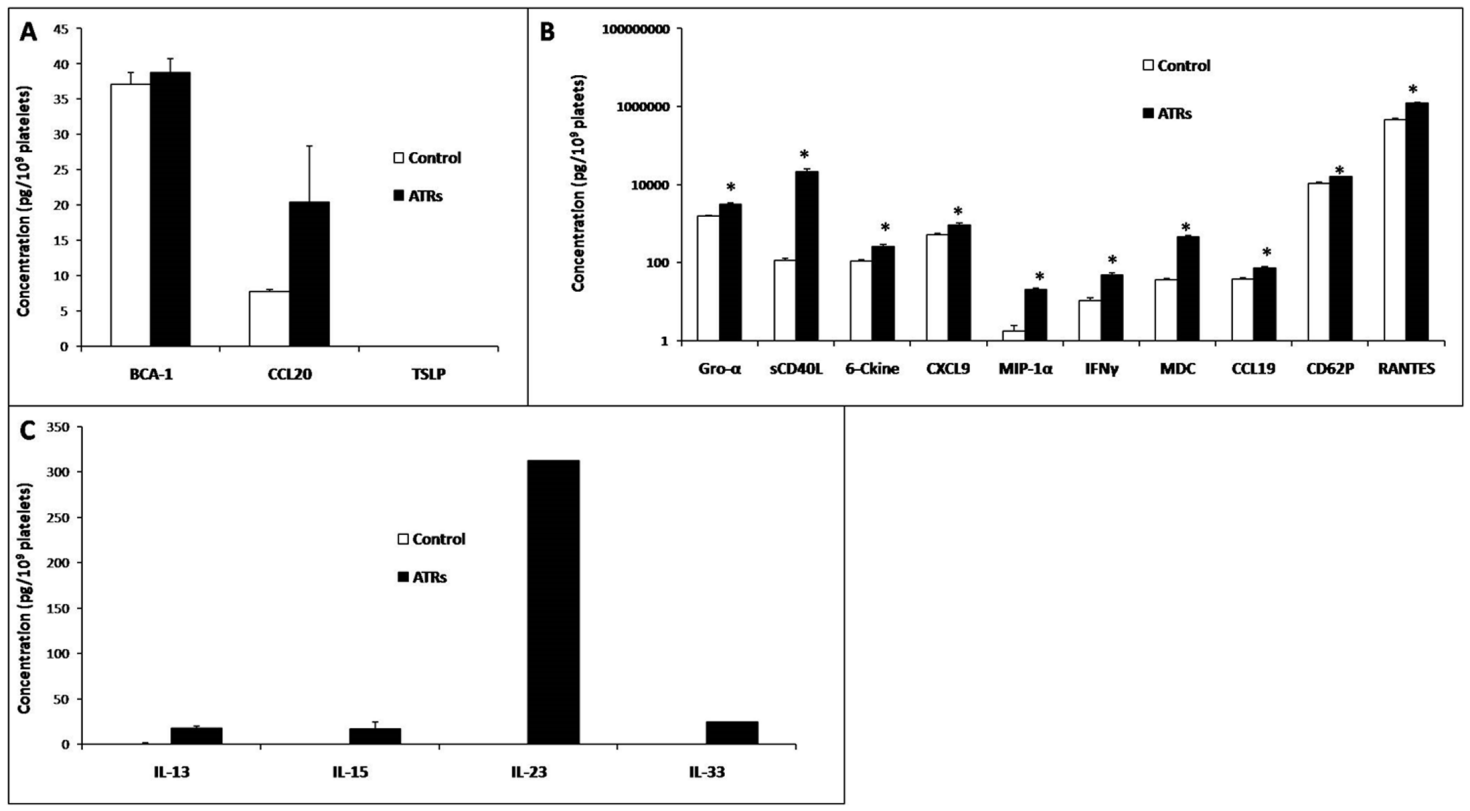
Concentrations of 17 soluble factors in the supernatants from 65 ATRs PCs and 59 control PCs. The data are adjusted to pg/10^9^ platelets and expressed as the mean ± SEM. **A**. Factors that did not display any difference between the control and AE samples. **B**. Factors that had a concentration in the AE samples that was significantly higher than in the control samples or that were detected only in the ATR samples. **C**. Factors that were not detected in the controls, regardless of the amounts in the ATR samples (concentrations in the control and ATR samples were compared using two-tailed Student's t test, *p<0.05).

We initially observed three types of responses. 1) Three factors were not relevant in this series, either because they were equally present in PCs that produced ATRs and in controls (with high consistency, e.g., BCA-1, or with high variability precluding homogeneity and significance, e.g., CCL20) or because they were absent in both types of SDA-PCs (e.g., TSLP) (**Fig. 2A**). 2) Ten of these factors were significantly (p<0.05) more elevated in SDA-PCs that produced ATRs in the recipients than in the matched control PCs. In order from maximum to minimum elevated amounts, these factors were RANTES, sCD62P, sCD40L, Gro-1α, CXCL19, C-CKine, MDC, IFN-γ, and CCL19 (**Fig. 2B**). 3) Four factors were classified within the “pathogenic-type” PCs and were not detectable in the controls, even though the amounts in the ATR-inducing PCs were higher than trace amounts, i.e., there were order-of-magnitude differences: IL-23, IL-33, IL-13, and IL-15 (**Fig. 2C**). Importantly, to the best of our knowledge, none of these BRMs have been previously included within the commonly acknowledged platelet-associated molecules [30].

### Relevance of platelet-associated immunomodulatory factors in the stored platelet components (transfusion inventory)

Several years ago, we and others produced evidence that platelets stored from day 1 (after collection and processing, standing for incoming in distributable products, i.e., the inventory) until days 5 to 7 were capable of secreting copious amounts of BRMs, independent of any deliberate addition of activation factor(s). The secretion profile of many of these BRMs appears to depend solely on the shelf-life of the platelets, [2], [15], [20],[21],[31],[32], but it may be modified by the initial (whole blood buffy-coat pools vs. SDA-PCs) or additional processes (PAS vs. 100% autologous plasma) [33]. The profile also depends on the type of cell separator when considering SDA-PCs [33]. Herein, because we identified a number of BRMs that have not previously had their secretion kinetics in shelf-life storage evaluated, we sought to evaluate their profiles between days 0 and 5 after collection to determine any possible relevance with the present case study. We considered the amount of each cytokine in the remaining PC returned to our BE facility and compared the BRMs in the “pathogenic” and non-pathogenic (control) PC bags.

**Figure 3:**
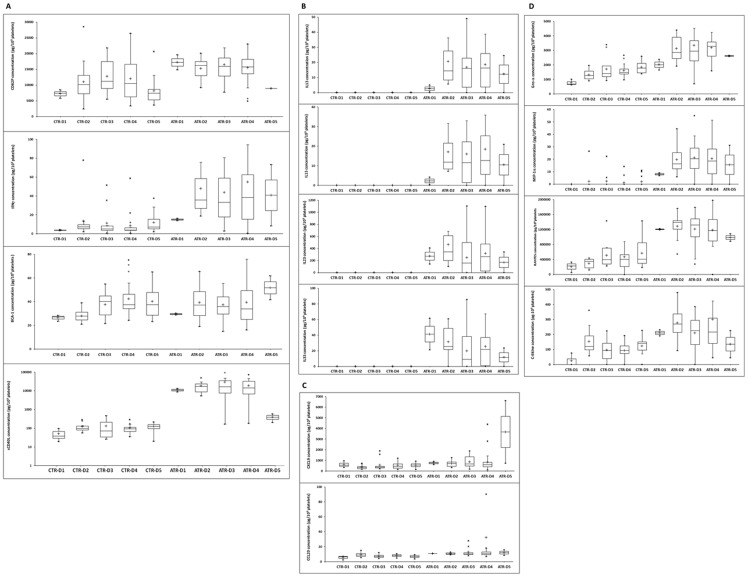
Release of soluble factors during platelet storage for 5 days. The data are adjusted to pg/10^9^ platelets and expressed as the mean ± SEM. **A**. Factors that increased over 5 days of storage in only the control samples. **B**. Factors that were constantly secreted with almost equivalent amounts between days 1 and 5 in the supernatants from “pathogenic” PCs but that were not detectable at any time between days 1 and 5 in the control samples. **C**. Factors with invariable trace amounts—between days 1 and 5 in the control PC supernatants and with elevated concentrations, but only on days 4 and 5, in the “pathogenic” PC supernatants. **D**. Factors that were elevated in both the control and pathogenic supernatants, although with significant variations between the control and ATR samples (concentrations of the soluble factors on days 2–5 vs. day 1 in the same group were compared using ANOVA, *p<0.05).

In addition, we identified four main profile types. 1) Five BRMs (RANTES, sCD62P, BCA-1, IFN-γ and CCL19) were consistently secreted by both “pathogenic” and control PCs, with no significant change over time from days 1 to 5. However, although there appeared to be no variation at all between either situation regarding BCA-1, the amounts of sCD62P, sCD40L, BCA-1, and IFN- γ were slightly (the former three) or significantly (the latter; p<0.05) more elevated (**Fig. 3A**). 2) Another four BRMS (IL-23, IL-33, IL-15, and IL-13) were constantly secreted in almost equivalent amounts from days 1 to 5 in the supernatants from “pathogenic” PCs, whereas they were not detectable at any time from days 1 to 5 in the control samples (**Fig. 3B**). 3) Similar to the previous situation, CCL20 and CXCL9 were found at trace, although quite invariable, amounts between days 1 and 5 in the control PC supernatants and at elevated concentrations in the “pathogenic” PC supernatants on days 4 and 5 (p<0.05) (**Fig. 3C**). The decrease in CCL20 on day 5 may be attributable to whole protein degradation, which prevented optimal access to the detecting Ab, as has been observed in previous studies [2], [34]. The last four BRMs (Gro-α, RANTES, MIP-1α, and C-6kine) (**Fig. 3D**) were elevated in both the control and “pathogenic” supernatants, although with significant variations between the controls and ATR cases (a finding that was not unexpected for sCD40L as it confirms our and others' findings) [6], [14], [16], [21].

**Figure 4:**
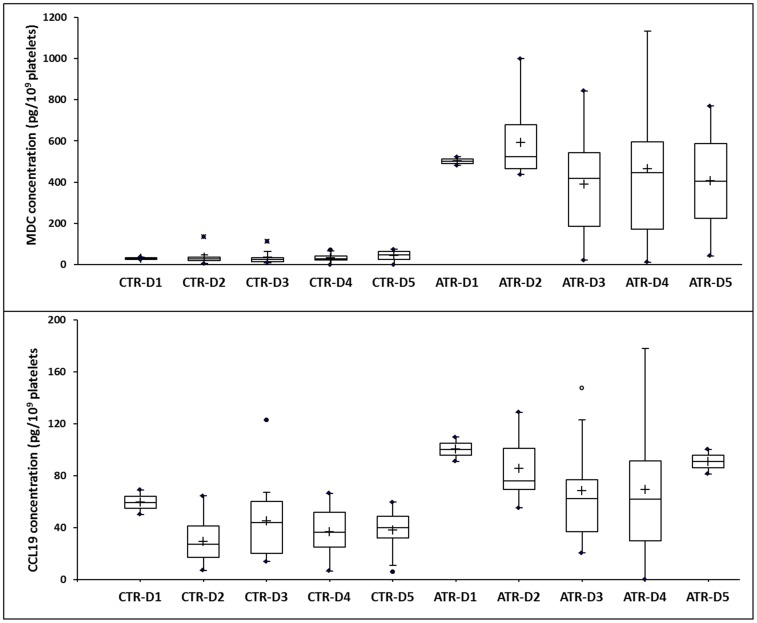
Factors that displayed no significant modulation in both the control and ATR samples during storage.

The control PCs were prepared just as they would be for transfusion purposes, with the exception that small volumes were sampled at the time of delivery for this study. We observed no significant modulation of the CCL22 (MDC) and CCL19 concentrations in the platelet supernatant during storage (**Fig. 4**). The levels of sCD40L and sCD62P increased notably by day 3 (on average by 160% and 77%, respectively), and the levels of 6-Ckine, RANTES, and Gro-α increased by day 5 (on average by 385%, 138%, and 238%, respectively). In contrast to the “pathogenic” PC supernatants, we observed no modulation of platelet immunomodulatory factor concentration during storage, most likely because of an initially high concentration of this molecule. This finding most likely suggests that a hyperresponsive platelet status characterizes the PCs involved in ATRs (**Fig. 2C** **and** **Fig. 3B**).

### Platelet components associated with acute transfusion reactions display characteristic profiles of secreted platelet factors

The present study revealed that among the various BRMs tested—even though the series was not comprehensive—there were preferential associations of secreted products (**Table 2**). For example, sCD40L was significantly associated with (p<0.05 with α = 0.05) MIP-1α (Pearson's correlation coefficient PCC = 0.56), IL-13 (PCC = 0.472), IFN-γ (PCC = 0.461), IL-15 (PCC = 0.545), MDC (PCC = 0.336), and sCD62P (PCC = 0.549) but not with 6-Ckine, CXCL9, IL-23, IL-33, CCL19, RANTES, CCL20, and BCA (NS). In contrast, IL-33, an alarmin-like cytokine that usually characterizes endothelial and epithelial cells [35], has not been previously reported in association with platelets. IL-33 was found in the present study to be significantly correlated with 6-Ckine (PCC = 0.649), IL-23 (PCC = 0.707), MDC (PCC = 0.277), and CCL19 (PCC = 0.365).

**Table 2:** Pearson correlation matrix of 16 soluble factors in 65 ATRs supernatants.

Factors	Gro α	sCD40L	6-Ckine	CXCL9	IL23	MIP-1α	IL13	IFNγ	IL15	MDC	IL33	CCL19	CD62P	RANTES	CCL20	BCA
Gro α	**1.00**															
sCD40L	**0.85**	**1.00**														
6-Ckine	−0.05	0.02	**1.00**													
CXCL9	0.09	0.04	−0.10	**1.00**												
IL23	−0.08	−0.07	**0.51**	−0.13	**1.00**											
MIP-1α	**0.52**	**0.56**	0.05	−0.11	0.14	**1.00**										
IL13	**0.39**	**0.47**	0.07	−0.09	0.22	**0.91**	**1.00**									
IFNγ	**0.44**	**0.46**	0.06	0.02	0.16	**0.91**	**0.85**	**1.00**								
IL15	**0.46**	**0.55**	0.09	−0.09	0.16	**0.90**	**0.95**	**0.88**	**1.00**							
MDC	**0.45**	**0.34**	0.13	0.08	**0.29**	**0.57**	**0.50**	**0.60**	**0.45**	**1.00**						
IL33	−0.08	−0.02	**0.65**	−0.12	**0.91**	0.08	0.16	0.09	0.13	**0.28**	**1.00**					
CCL19	0.06	−0.02	**0.39**	0.20	**0.36**	−0.07	−0.02	0.01	−0.08	**0.48**	**0.36**	**1.00**				
CD62P	**0.56**	**0.55**	0.07	0.04	0.13	**0.40**	**0.30**	**0.34**	**0.35**	**0.53**	0.19	0.22	**1.00**			
RANTES	0.12	0.15	0.12	−0.14	−0.06	**0.27**	**0.33**	**0.27**	**0.32**	0.17	0.02	0.04	0.16	**1.00**		
CCL20	0.05	−0.07	−0.06	0.03	−0.07	0.17	−0.07	0.19	−0.07	−0.02	−0.15	0.01	−0.10	−0.10	**1.00**	
BCA	0.22	0.09	0.13	0.21	0.17	**0.29**	**0.34**	**0.35**	**0.30**	0.22	0.11	**0.36**	0.10	0.10	**0.31**	**1.00**

The bold values were different from 0 at a significance level of α = 0.05.

### The selective content in secreted factors present in platelet components may be predictive of the risk of an acute transfusion reaction

We next aimed to determine whether given profiles of BRMs, which would have significant associations in certain PCs producing ATRs, were random. Thus, we evaluated the frequency of each product in the each of the 65 cases and its association with the other 64 cases using the ROC method, which allows the estimation of the chances that a given product is present stochastically and not specifically.

**Figure 5:**
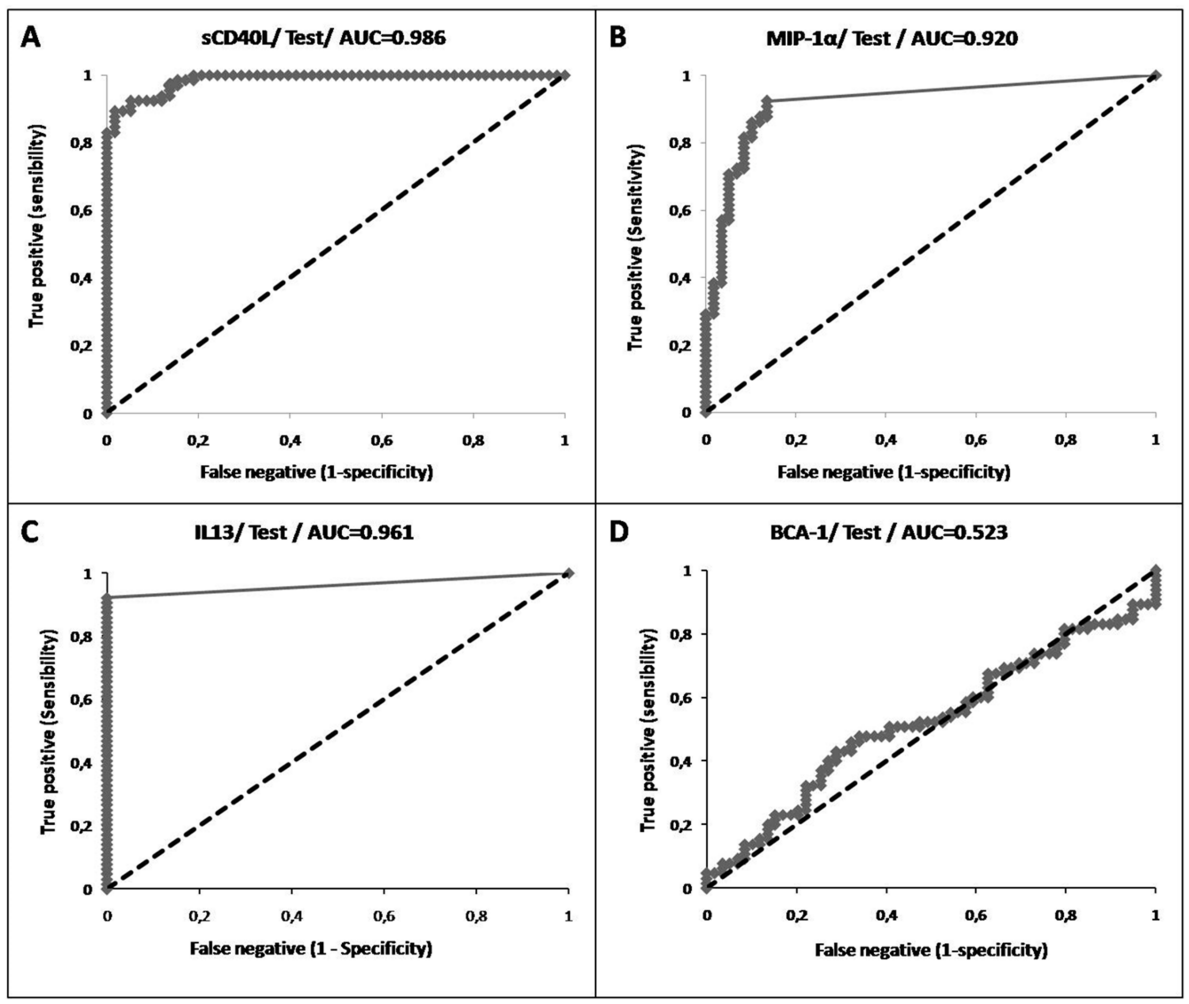
Examples of areas under the ROC curves. **A**. sCD40L; **B**. MIP-1α; **C**. IL13; **D**. BCA-1.

A ROC curve of each soluble factor was generated and displayed the relationship between the fraction of true positives (i.e., a specific increase in the considered soluble factor concentration in “pathogenic” PC supernatants) and the fraction of false positives (i.e., an increase in the considered soluble factor concentration in supernatants that were not reported to be associated with an AE) at various threshold settings. The area under the curve (AUC) of a factor was then used to illustrate its performance to classify the PCs into two categories (control or ATR-associated) and to select the best cut-off values for the cytokines/BRMs in the PC supernatants associated with ATRs. The greater the importance of the discriminatory ability, the more the ROC curve deviates from the random classifier line and becomes closer to the ideal classifier line. For example, sCD40L (**Fig. 5A**), MIP-1α (**Fig. 5B**), and IL-13 (**Fig. 5C**) were better predictive markers for the possibility of the occurrence of an ATR in PC supernatants consisting of these molecules than the other BRMs, even if the latter were associated with ATR cases (e.g., BCA-1) (**Fig. 5D**).

We next calculated the AUC, which represents the level of prediction of a given factor regarding the possible occurrence of an ATR. The discriminatory ability of each factor was compared with 0.5 (random classifier), with a p-value <0.0001 (z-test). When the AUC was different from 0.5, the ATR occurrence was likely not random. Platelet supernatant levels with significant levels of sCD40L, IL-13, MIP-1α, RANTES, Gro-α, MDC, IL-15, CCL20, IFN-γ, 6-Ckine, CCL19, sCD62p, and CXCL19 displayed significant association with the occurrence of an ATR compared with the control group. In contrast, the levels of cytokines and BRMs such IL-33 and IL-23 proved less sensitive, and BCA-1 was not informative at all, being similar to the controls (**Table 3**). The cut-off value of each soluble factor test with the optimal sensibility and specificity is presented in **Table 3**.

**Table 3:** Discriminatory ability of soluble factors to classify PCs as belonging to either the adverse effect or control group.

Factors	AUC	p	Threshold pg/10^9^ plt
**sCD40L**	0.986	<0.0001	289.5
**IL13**	0.961	<0.0001	0
**MIP-1α**	0.92	<0.0001	9.9
**Rantes**	0.917	<0.0001	877638.3
**Gro α**	0.917	<0.0001	2571.6
**MDC**	0.916	<0.0001	70.6
**IL15**	0.915	<0.0001	0
**CCL20**	0.857	<0.0001	9.6
**IFNγ**	0.854	<0.0001	18.1
**6-Ckine**	0.807	<0.0001	150.1
**CCL19**	0.762	<0.0001	53.69
**CD62P**	0.755	<0.0001	13126.9
**CXCL9**	0.692	<0.0001	535
**IL23**	0.875	0.003	
**IL33**	0.389	0.0004	
**BCA - 1**	0.523	0.655	

All AUCs with a p-value of <0.0001 (z-test) were considered different from 0.5.

In aggregate, when the data for individual cases are cross-sectioned for prediction based on ATR cases with the random data possibly found in the control cases, few platelet-associated soluble factors (parameters) are reliably predictive of an ATR outcome if considered in isolation.

### Results from “learned decision trees”

**Figure 6:**
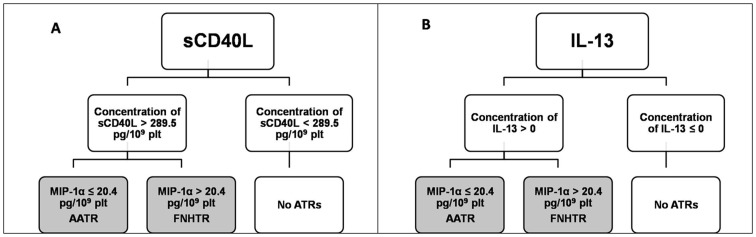
Decision tree. **A**. Assays without IL13 (among 16 assays, the success rate of the sCD40L model was the highest, 78%); **B**. Assays with IL13 (among 17 assays, the success rate of the IL13 model was the highest, 82%).

To obtain a descriptive, interpretive model of the functional relationship between a given set of cytokines selected by a cross-validated committee method, we applied a decision tree with the Weka platform. A decision tree describes several paths leading to leaves that assign a class to a new case, such as that depicted in **Fig. 6**. Given an individual donor PC's BRM profile, a two-branch decision may be designed downward to one of the two terminal nodes (ATR and control boxes). We can thus predict that an ATR is excluded when sCD40L ≤289.5 pg/ml/10^9^ platelets. In contrast, if the PC sCD40L level is >289.5 pg/ml/10^9^ platelets, there is a significant risk of an ATR. Interestingly, the model further predicts that when sCD40L is >289.5 pg/ml/10^9^ platelets and the MIP-1α level is >20.4 pg/ml/10^9^ platelets, there is a significant risk of an FNHTR-presenting ATR. In the presence of a similar amount of sCD40L (>289.5 pg/ml/10^9^ platelets) and when MIP-1α is ≤20.4 pg/ml/10^9^ platelets, there is a significant risk of an AATR-presenting ATR (**Fig. 6A**). Surprisingly, given that the involvement of sCD40L in a transfusion-like pathology was not a surprise to us, another BRM, unexpected in this role at that time, proved highly informative. On the decision tree model based on IL-13 values, this secreted product was demonstrated to be equivalent to sCD40L in predicting an ATR occurrence. When IL-13 is ≤0 pg/ml/10^9^ platelets, the risk of ATR was very unlikely, but when IL-13 is >0 pg/ml/10^9^ platelets (i.e., in the presence of even minute amounts of this interleukin) and MIP-1α is >20.4 pg/ml/10^9^ platelets, then there is a significant risk of an FNHTR-presenting ATR. Conversely, when IL-13 is >0 pg/ml/10^9^ platelets and MIP-1α is ≤20.4 pg/ml/10^9^ platelets, then there is a significant risk of an AATR-presenting ATR (**Fig. 6B**). These data are valuable because one can theoretically deduce the possibility of a risk attributable to a given PC at both the time of its administration and the clinical presentation. However, the application of these findings is not currently practical for daily clinical practice. Even if these findings are meaningless in practical clinical medicine, they reinforce the idea that platelets have cytokine/BRM secretion programs that are not merely stochastic but also instrumental in altering the recipient's physiology and eventually facilitating pathology.

## Discussion

Despite transfusion has become extremely safe thanks to additional safety measures overtime, the remaining risks fall chiefly into three main categories: overload and metabolic accidents (and—at large—the technique of transfusion), human errors (comprising chain errors, such as the wrong product to the wrong patient), and an emerging hazard characterized by deleterious inflammation in the patient (recipient). Among the transfused LBCs, PCs lead to nearly half of the reported AEs, whereas they account for only 10% of the BCs. PCs are also the BC that leads to the majority of inflammation-associated AEs and ATRs in aggregate. One likely explanation is that platelets continuously secrete several hundred collectively termed BRMs. Based on their *ex vivo* observations, several groups, including ours, harbored the suspicion that such pro-inflammatory factors are related to the reported inflammatory symptoms in ATRs, although directly relating the clinical and laboratory findings was not possible. Our reported observations concerned indeed patients suffering from onco-haematological or oncological diseases and having undergone radio-chemotherapy. Surgical patients were not considered here because they are mostly transfused in emergency; they may have received several LBCs by the same time, including large volumes of plasma, and – regarding PCs – we could not assume in those cases the full respect of ABO plasma and cell compatibility. Onco-hematological patients surveyed here were ABO matched in a about 80–85% of the cases—plasma compatibility (cell compatibility case about 95% for such patients)—because this issue is taken as a serious quality measure by the BE in charge; it cannot be excluded however, that in the remaining 15% patient cases, some so called “minor” ABO incompatibility has played a role in the reported inflammatory process. In accordance with studies by others—addressing other questions on separate parameters—we observed (**Fig. S2**) that either platelet separator used here (Trima-Accel and Amicus) led to comparable levels of pro-inflammatory BRMs; either separator is considered equal for quality control of platelets and corrected count increment studies [36], [37].

This report is most likely the first to offer direct evidence that some PCs contain platelets having differential secretion capacity without pre-identified activation signal(s). As some sets of BRMs may favor a pathogenic situation in certain types of recipients, this work demonstrates, on the one hand, that secreted BRMs are not stochastic, but rather display significant associations, and on the other hand that some BRM associations are instrumental for triggering an inflammatory response in the recipient when their gross amounts exceed a threshold. They are but also critical in the clinical manifestation of the ATR symptoms. Because we aimed to decipher the conditions that render a PC potentially able to produce an ATR, we developed a statistical model that predicts the safety or pathogenicity of a given PC. This application is a first in transfusion medicine, a discipline that makes every effort to dampen the unavoidable mismatched characteristics of blood donors and recipients and their consequences; however, these efforts are based only on adapted immune parameters (blood group and human leukocyte antigen/human platelet antigen [HLA/HPA] antigens) that can be grossly tested or cross-matched by serology. We have now extended the safety outcomes of BC compatibility to innate immunity while keeping in mind that inflammation can be extremely serious and life threatening.

In more detail, the present work is novel in at least three ways. First, it describes six novel cytokines that were not known to have a “normal” association with platelets, even though more than 1,000 proteins have been previously associated with platelet functions and physiology, including 626 that can be secreted [38]. These six cytokines should be added to another two that our group recently identified (IL-27 and Ox40L) that are also associated with transfusion pathology in a pathogenic triad (95%) with sCD40L [23]. These findings indicate that the platelet world is far from being completely understood. The reason why some platelet (glyco)-proteins are misrecognized is most likely—as has been acknowledged in this report—because they are only essentially revealed in pathological conditions. IL-13, for example, appears pathogenic when it is highly secreted. This cytokine is not borrowed from the donor's plasma (as the detection level in normal plasma is under the minimum level of detection of IL-13 with our method [0 pg/ml]), and as shown here, IL-13 can be elicited upon appropriate *in vitro* stimulation (as described in an allergic asthma context) [39]. Whether IL-13 (and other pathogenic-type cytokines) is secreted by platelets in certain PCs without an apparent stimulus, while those PCs are maintained under generally suitable sterile conditions that are used worldwide, remains to be discovered. Although, at this time, it is highly speculative, we favor the donor genetics hypothesis (certain donors may present with characteristics that render them prone to stimulate certain BRMs upon lower stimulus thresholds than the general donor population). This characteristic remains at a physiological level until those platelets are infused to an unrelated individual (a patient/recipient) because they constitute a PC for transfusion purposes.

The second level of novelty in this work is that it offers insights into platelet-linked pathologies. Previous reports have revealed that platelet-secreted products become associated when they are stimulated. We recently extended data that were initially generated using *in vitro* models to clinical situations involving transfusion. Nearly all ATR cases tested (29/30), for example, relate to PCs with elevated levels of sCD40, IL-27, and Ox40L together. A general difficulty in transfusion hazards is relating *ex vivo* observations to clinical issues. The present report, with a “yes or no” situation for IL-13, provides such direct evidence. Furthermore, the MIP-1α data demonstrate a clear relationship with the symptoms, which was not expected. MIP-1α is produced by T and B cells, Langerhans cells, neutrophils, and macrophages and is also produced by platelets (α granules) [30]. MIP-1α has proinflammatory activities involving the attraction and activation of leukocytes at large [40]. Several reports indicate an intimate relationship between leukocyte-endothelial cells, adhesion molecules, and the expression of the monocyte-derived chemokine MIP-1α during cellular adhesion. This mechanism may serve an important role in cell activation and the recruitment of leukocytes during the initiation of an inflammatory response [41]–[43]. Platelet-originating factors (BRMs) are known to be involved in transfusion pathology. sCD40L, a cytokine-like product that originates essentially from platelets (almost 95% [44]), has been associated with pathology in *ex vivo* models. Furthermore, Silliman et al calculated that sCD40L at 10 ng/mL was able to trigger TRALI experimentally. Nevertheless, more recent data from Toy et al dispute the causative role of sCD40L in the physiopathology of TRALI. Our present data do not address the TRALI issue but do confirm the pathogenic role of sCD40L in transfusion above a certain level (exceeding 289.5 pg/10^9^ platelets herein). In addition, platelet-issued products such as MPs have been demonstrated to alter tissues, such as joints [45]. In aggregate, these data suggest balanced roles of platelet products, being physiologic or pathologic depending on the stimulation mode, the amount secreted, and the site of secretion. Our data contribute to this knowledge base with an ATR prediction model. This model relates directly to transfusion safety. Overall, much progress has been achieved in transfusion safety. Transfusion transmitted infectious risks have been minimized by improving the medical selection of donor candidates and biological testing of donated blood. Many BEs also perform bacterial testing with PC delivery. Immunological risks have been reduced by baseline or extended immuno-hematological testing to prevent Ag/Ab conflicts. Inflammation is addressed in transfusion safety essentially by measures to avoid three types of hazards also linked with an Ag/Ab conflict: ABO mismatches (which create a potentially lethal cytokine storm), TRALI, and severe allergic reactions. Moreover, allergic reaction is most commonly due to infusion of plasma proteins, may occur in up to 1% of all transfusions and often seen with FNHTR. However, the potential hazard of individual BCs is not currently addressed, except for bacterial detection, but the current techniques have many limitations. The avoidance of anti-HLA Abs in BCs is generic and not adjusted to specific patient situations. Our approach proposes a new paradigm in transfusion medicine. It postulates that PCs can create risk independent of the immunization process. Those risks are unpredictable by current means. Because transfusion, particularly PC transfusion, is intended to treat fragile patients, avoiding any additional risk for those patients and making an effort to ensure the safest BC are legitimate.

Third, this work provides new insight in translational medicine. It proposes the use of statistical tests to assist decision-making to avoid hazards. In contrast to other disciplines in which a ‘yes or no’ decision has to be made to avoid the secondary effects of a given drug, in this case, the math offers the possibility to select the best-fitted treatment for a given patient and to discard BCs that may be at risk. Although the risk is not certain, based on the principle of precaution and until novel decision trees are created to decipher who is an at-risk patient, the decision of not considering these BCs for use in patients, especially in the most fragile patients who have a minimal ability to cope with acute inflammatory syndrome, may be made.

In aggregate, the present model does not as yet assist delivery for all patients awaiting PC transfusion. Doing so would threaten the PC inventory and considerably delay administration, a situation not compatible with current emergency needs. Furthermore, the model is perhaps not optimal because it cannot be excluded that other BRMs, which were not tested here, are not more relevant than those that were tested, and therefore selected, here. Finally, the model would be completed by a test that also predicts who may be a patient at risk of presenting post-transfusion acute inflammation. As it stands, the model facilitates moving toward the design of better-fitted assistance to patients, which can use prediction tests provided by professional statistical models.

Machine learning approaches have wide applications in bioinformatics, and decision trees are one of the most popular and successful approaches applied in this field [46]. Based on a large number of assays (17 in this study) with limited sample sizes (n = 124), this method allows the generation of a simple, interpretable, and reliable model such as a model using IL-13 or sCD40L. Either model can distinguish pathological PCs from the control PCs with a high success rate. In medical or biomedical research, this approach is used more often for disease prediction and screening. One of the major challenges for proteomic studies is the comprehension of mining biologically useful information from the *in vitro*, *ex vivo*, or *in vivo* data. In addition, non-classical statistical methods for data analysis need to be performed. To obtain a comprehensible picture of biological phenomena at the molecular, cellular, and organismal levels, researchers must evaluate both all of these attributes and the relationships among them. Therefore, various machine learning classification algorithms have been developed for biological data analysis, including decision trees [47], [48]. The decision tree algorithm appears to poorly be used in the transfusion context as blood involves a large amount of biological data involving intricate parameters from i) the donor, ii) the labile blood components and processing attributes, and iii) the recipient [49].

## Supporting Information

Figure S1 Study design for PCs delivered at EFS Auvergne, Loire, France.
(TIF)

Figure S2 Differences in some soluble factor concentrations in the control PCs prepared by the Trima and Amicus processes during storage. The concentrations of soluble factors in PC supernatants at days 2–5 vs. day 1 following preparation with the TRIMA process and at days 3–5 vs. day 2 with the Amicus process were compared using ANOVA, *: p<0.05 respectively. #: Significant difference in the concentration of soluble factors between the TRIMA and Amicus processes on the same day (t test, p<0.05).
(TIF)

Methods S1 Single donor platelet component preparation, delivery and surveillance.
(DOCX)
