# Supplementary material for: A Computerized Prediction Model of Hazardous Inflammatory Platelet Transfusion Outcomes
Source: PLoS One. 2014 May 15;9(5):e97082. doi: 10.1371/journal.pone.0097082 (PMC4022636; doi:10.1371/journal.pone.0097082)
Supplement: Methods S1 — Single donor platelet component preparation, delivery and surveillance. (DOCX) [file pone.0097082.s003.docx]

***Supplemental Materials and Methods***

***Single donor platelet component preparation, delivery and surveillance***

Blood was collected on acid citrate dextrose-A with Amicus (Software Version 2.5, Fenwal, Inc., Lake Zurich, IL) or Trima (Gambro BCT, Lakewood, CO) cell separators. The blood anticoagulant ratio was 10:1. The mean target level of the platelets was 5.26 ± 0.6 × 10^11^ to prepare two identical components in PL2410 containers (Fenwal-Europe, La Châtre, France). All collection procedures were performed in one of the two regional authorized settings. The samples were immediately shipped to the unique processing platform of this BE and distributed after qualification, which was generally between 3 and 5 days after collection (>75% by days 4 and 5). The preparation procedure and the incident/accident declaration strictly conformed to the national protocols, as described in the previously reported procedure [[1](#_ENREF_1)]. Following the occurrence of an AE, immediately after declaration, the incriminated PC bags were shipped back the BE facility for immediate processing. If it was an overnight AE, the processing time never exceeded 12 h.

Platelets were sampled at the delivery timepoint for control PCs or at the time of incident declaration for the AE PCs for evaluation of an array of *in vitro* properties subsequent to thrombin receptor activator peptide (TRAP; peptide SFLLRN) stimulation, which we used as a positive control (Sigma-Aldrich, Saint Quentin-Fallavier, France; 50 µg/ml).

The quality control parameters were as follows: mean volume of PC bags (372 ± 36 ml); platelet counts by automated blood cell counter (ABX-ARGOS, Abx Diagnostics, Montpellier, France) (mean platelet count of 5.31 ± 0.65 × 10^11^ platelets/PC); applying the leukoreduction process to all PC bags to maintain a residual white blood cell (WBC) count of less than 10^6^ per unit (the number of residual WBCs was counted by flow cytometry using a WBC counting kit: LeucoCOUNT, Becton Dickinson, Le Pont de Claix, France) (mean residual leukocyte count of 0.095 ± 0.087 × 10^6^ leukocytes/PC); and a mean pH that remained constant until the time of delivery to the patient (pH 7.3 ± 0.1). All PCs were automatically re-suspended in 35% autologous donor plasma and 65% platelet additive solution (PAS: InterSol™, Fenwal, la Châtre, France or SSP^+^™; MacoPharma, Mouveaux, France) and stored at 22 ± 2°C with gentle rotation/shaking (60 rpm) for a maximum of 5 days before being issued for transfusion.

In the literature, Burgstaler EA et al., showed that the Trima Accel machine processed significantly more whole blood with equivalent PLT yields, processing time, and number of PLTs per minute compared to the Amicus single-needle procedure, but had a significantly lower collection efficiency [[2](#_ENREF_2)]. Moreover, Fontana S et al. described that both cell separators showed safe collection of up to 4 PLT units per donation with adequate corrected count increments. Gambro BCT Trima Accel Version 5.0 produced a higher platelet yield despite shorter apheresis duration, but with slightly higher residual WBC counts and a trend for higher side-effect frequency [[3](#_ENREF_3)]. Our internal control during the years 2008 to 2011 showed that the mean residual leukocyte counts were not significantly different and corresponded to 0.095 ± 0.087 and 0.109 ± 0.1× 10^6^ leukocytes/PC), respectively for Amicus or Trima cell separators.

**References:**

1. Chavarin P, Cognasse F, Argaud C, Vidal M, De Putter C, et al. (2011) In vitro assessment of apheresis and pooled buffy coat platelet components suspended in plasma and SSP+ photochemically treated with amotosalen and UVA for pathogen inactivation (INTERCEPT Blood System). Vox Sang 100: 247-249.

2. Burgstaler EA, Winters JL, Pineda AA (2004) Paired comparison of Gambro Trima Accel versus Baxter Amicus single-needle plateletpheresis. Transfusion 44: 1612-1620.

3. Fontana S, Mordasini L, Keller P, Taleghani BM (2006) Prospective, paired crossover comparison of multiple, single-needle plateletpheresis procedures with the Amicus and Trima Accel cell separators. Transfusion 46: 2004-2010.

***Supplemental Figure 1***

***
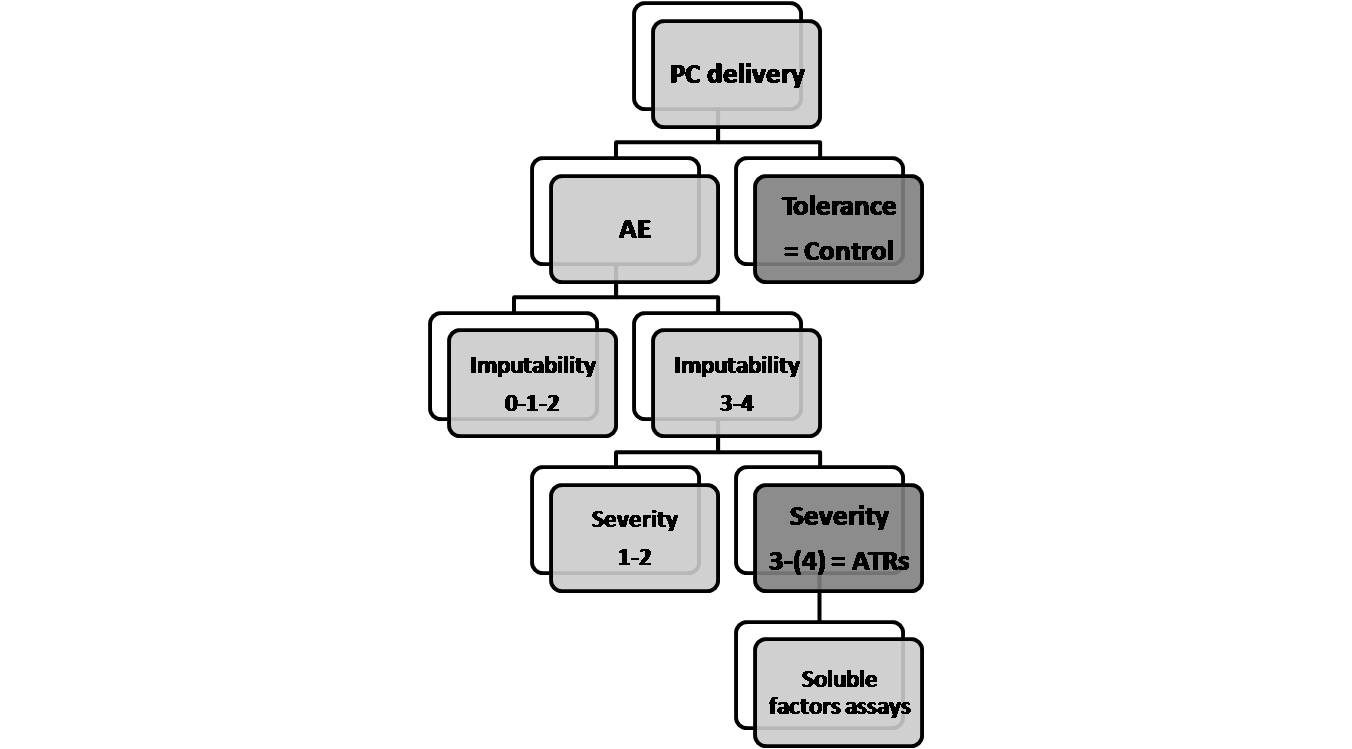
***

***Supplemental Figure 2***

***
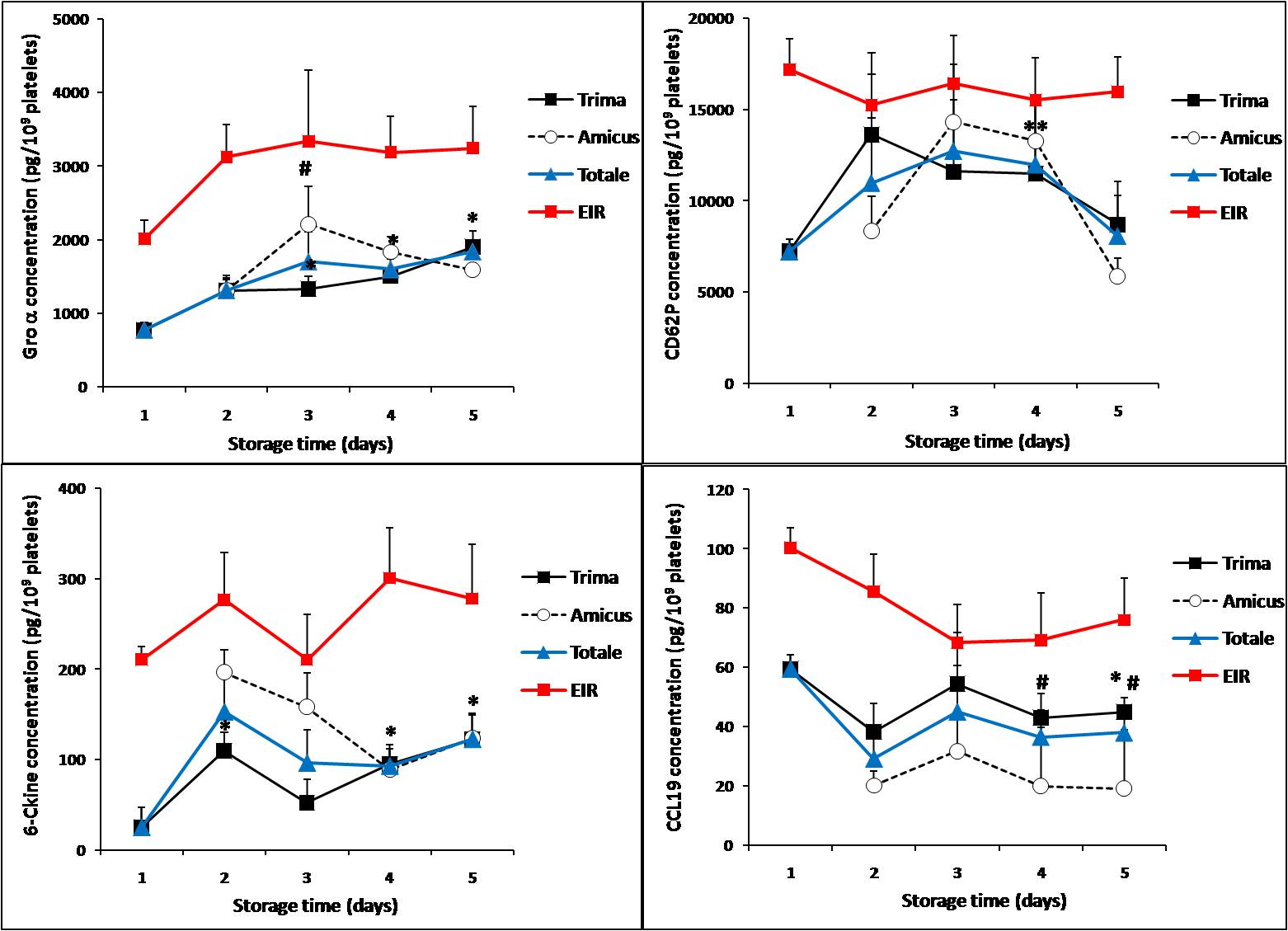
***
